# A calcium-dependent protein kinase, ZmCPK32, specifically expressed in maize pollen to regulate pollen tube growth

**DOI:** 10.1371/journal.pone.0195787

**Published:** 2018-05-29

**Authors:** Jie Li, Yihao Li, Yanling Deng, Ping Chen, Fen Feng, Wanwan Chen, Xiaojin Zhou, Yingdian Wang

**Affiliations:** 1 Beijing Key Laboratory of Gene Resources and Molecular Development, College of Life Sciences, Beijing Normal University, Beijing, China; 2 Department of Crop Genomic & Genetic Improvement, Biotechnology Research Institute, Chinese Academy of Agricultural Sciences, Beijing, China; Institute of Genetics and Developmental Biology Chinese Academy of Sciences, CHINA

## Abstract

Calcium-dependent protein kinases (CPKs) play an essential role in the regulation of pollen tube growth. Although *CPK* genes have been identified in maize, and some have been functionally characterized, the molecular function of ZmCPKs associated with pollen tube development remains less well studied. Here, we report that a pollen-specific CPK, ZmCPK32, is involved in the regulation of pollen germination and tube extension. ZmCPK32 exhibited CPK activity and was localized on the plasma membrane and punctate internal membrane compartments via N-terminal acylation. *In situ* hybridization and real-time PCR revealed that *ZmCPK32* transcripts accumulated in pollen and expression was dramatically upregulated during shedding. To elucidate the function of this gene, we transiently expressed a *ZmCPK32-GFP* fusion protein in tobacco pollen using microparticle bombardment. ZmCPK32 accumulation inhibited pollen germination and reduced pollen tube growth, but this effect was abolished when the kinase-inactive variant was expressed, indicating that kinase activity is critical for its regulatory function. In addition, the plasma membrane localization of ZmCPK32 is essential for regulating polar growth, as pollen expressing the cytosol-localized kinase displayed reduced tube length but germinated well. Moreover, the constitutively active form of ZmCPK32 enhanced the reduction in the germination rate, indicating that the specific activation of ZmCPK32 via calcium ions at the cortical growth point is essential for regulating appropriate germination. The results suggest that ZmCPK32 is functionally associated with pollen tube growth, and could represent a potential target for breeding male-sterile maize.

## Introduction

Pollen tube growth is important for plant reproductive development, as it delivers two sperm cells into the embryo sac. The pollen tube exhibits polar growth that depends on ion dynamics. Three cations, calcium (Ca^2+^), potassium (K^+^), and hydrogen (H^+^), and one anion, chlorine (Cl^-^), regulate pollen tube growth [1, 2], among which Ca^2+^ has a major role, since a tip-focused Ca^2+^ gradient is required for the polar growth of the pollen tube [3–5]. In addition, oscillations in tip Ca^2+^ concentration share the same frequency with the pollen tube growth rate [6–8], and tip localized Ca^2+^-dependent protein kinase (CPK or CDPK) activities are essential for polar pollen tube growth [5, 9, 10].

The Ca^2+^ signal can be sensed and transduced by a series of phosphorylation cascades regulated by various protein kinases, including CPKs, Ca^2+^/calmodulin-dependent protein kinases (CCaMKs), and calcineurin B-like interacting protein kinases (CIPKs). Among them, CPKs possess both a kinase and sensing domain, and include a variable N-terminal region, a kinase domain, an autoinhibitory junction domain, and a calmodulin-like domain (CaM-LD) with four EF-hand Ca^2+^-binding motifs [11, 12]. The N-terminal domain usually contains myristoylation and palmotylation sites, which are necessary for membrane localization. The junction domain serves as a pseudosubstrate that blocks the kinase active center in the absence of Ca^2+^, and detaches from the active center when Ca^2+^ binds to the CaM-LD [13]. Therefore, CPKs are considered to be sensor-independent kinases, because they contain both Ca^2+^-sensing (i.e., CaM-LD) and kinase domains, and can be activated by Ca^2+^ directly and transduce signals by phosphorylating substrates. By contrast, CCaMKs and CIPKs are sensor-dependent kinases, as their activation relies on the binding of Ca^2+^ sensors, such as calmodulin and calcineurin B-like protein.

CPKs, as an important node in Ca^2+^ signaling pathways, have been implicated in mediating Ca^2+^-regulated pollen tube growth [5, 9]. The essential role of CPK in promoting both pollen tube germination and growth was first identified in maize (*Zea mays*), as both germination and polar growth were impaired after inhibition of a pollen-specific CPK [9]. In addition, PiCDPK1 and PiCDPK2 have been reported to regulate pollen tube growth polarity and extension, respectively [14]. Furthermore, as a genetic evidence that CPKs are involved in pollen tube growth, *AtCPK17* and *AtCPK34* were shown to be essential for maintaining the pollen tube tip growth rate and facilitating the response to tropism cues [15]. Further research identified two pollen specific water and nonionic channels, NIP4;1 and NIP4;2, as substrates of AtCPK34 to regulate pollen germination and tube growth [16]. Showing an inverse correlation with the Ca^2+^ concentration, a negative gradient of anions at the pollen tube tip is also necessary for pollen tube growth, and is maintained by the anion efflux transporter S-type anion channel SLOW ANION CHANNEL-ASSOCIATED3 (SLAH3). CPK2 and CPK20 were shown to promote pollen tube growth by activating SLAH3 at the pollen tip [17]. Moreover, the *cpk11*/*24* double mutant exhibited enhanced pollen tube growth and impaired Ca^2+^-dependent inhibition of the inward K^+^ channels, suggesting that these CPKs negatively regulate pollen tube elongation. [18]. Taken together, these reports indicate that distinct CPK members may be associated with different aspects of pollen tube growth.

Maize is an increasingly important crop plant for food, feed, and industrial products, as well as a model monocot plant. Hybrid maize varieties are used extensively in modern farming, because the natural heterosis to hybrid maize provides increased yields and agronomic traits. However, hybrid maize seed production is laborious and time-consuming. Therefore, it would be beneficial to understand the mechanisms that regulate pollen tube development to provide potential approaches for breeding male-sterile lines. To date, progress has been made in elucidating the process of male gametophyte development in maize; however, less is known about the regulation of pollen tube polar growth, except for several studies that have provided clues into this process [19]. The protein Zm908p11, predominantly found in maize pollen, is expressed in mature pollen grains, and maize plants overexpressing this gene exhibited decreased pollen germination [20]. In addition, maize *aberrant pollen transmission 1* (*apt1*) was discovered by its impaired pollen transmission in heterozygotes and the formation of arrested pollen tubes. The *APT1* gene encodes a SABRE- and KIP-homologous protein with Golgi localization, suggesting a potential role in membrane trafficking, which is required for pollen tube elongation [21]. Recently, a phosphor-proteomic study revealed that many proteins with annotated functions in pollen germination are subject to phosphorylation regulation during pollen germination and tube growth. Moreover, many Ca^2+^-binding proteins and CPKs have been classified in the group of the most abundant phosphoproteins, suggesting that the CPK-mediated phosphorylation pathway is essential for pollen tube development [22].

In this study, we identified anther-specific *ZmCPK32* expression based on expression analysis of the maize *CPK* gene family. ZmCPK32 possesses CPK activity and is localized to the plasma membrane and punctate internal membrane compartments. Quantitative reverse-transcription (qRT)-PCR and *in situ* hybridization revealed that *ZmCPK32* accumulated predominantly in mature pollen grains, indicating it may be functionally associated with pollen tube development. We further explored the function of ZmCPK32 using transient expression in tobacco pollen. ZmCPK32 repressed pollen tube germination and growth, which depended on its kinase activity. In addition, the constitutively active form of ZmCPK32 could enhance the reduction in germination rate, while cytosol-localized ZmCPK32 weakly impaired pollen tube elongation. These results suggest that ZmCPK32 is functionally associated with pollen tube growth, and could represent a potential target for breeding male-sterile lines of maize.

## Materials and methods

### Plant material

The maize inbred line B73 and tobacco plants were grown in a greenhouse at Beijing Normal University (Beijing, China). All samples were frozen in liquid nitrogen and maintained at -80°C until use, except tissues for histochemical analysis, which were fixed immediately after collection.

### Expression analysis of maize *CPK* genes using RNA sequencing data

The fragments per kilobase per million mapped reads values of maize *CPK* genes for 79 distinct tissues were retrieved from the supplemental dataset of the maize gene expression atlas [23]. A heat map was constructed using Cluster ver. 3.0 and presented using TreeView software.

### Phylogenetic tree construction

The amino acid sequences of maize CPK proteins and those functionally associated with pollen tube growth were aligned using the ClustalX ver. 2.0 program. Then, a phylogenetic tree was constructed using the neighbor-joining method in MEGA ver. 4.0 software. The gene identifiers of maize CPKs are described in the results, and the proteins and accession numbers of other CPK proteins are described below: AtCPK2 (At3g10660), AtCPK11 (At1g35670), AtCPK17 (At5g12180), AtCPK20 (At2g38910), AtCPK24 (At2g31500), AtCPK32 (At3g57530.1), AtCPK34 (At5g19360), PiCDPK1 (DQ147913), and PiCDPK2 (DQ147912).

### Cloning of *ZmCPK32*

Total RNA was isolated from B73 maize tassel using plant TRIzol reagent (Invitrogen) according to the manufacturer’s instructions and treated with DNase I (Promega) to eliminate DNA contamination. Reverse transcription was performed using SS II (Invitrogen) according to the manufacturer’s protocol. To obtain the full-length cDNA of *ZmCPK32*, the primers CPK32-F and CPK32-R (S1 Table) were designed according to the sequence of the cDNA entry GRMZM2G332660 in MaizeGDB (https://www.maizegdb.org/). The PCR product was cloned into pGEM-T Easy (Promega) for sequencing.

### Plasmid construction

For mRNA *in situ* hybridization, the sequence containing the 3′-untranslated region of *ZmCPK32* was amplified with the primers ISH-F and ISH-R, and the resulting PCR fragment was cloned into pEASY-T3 (TransGen Biotech) to create the plasmid pEASY-Zm*CPK32ISH* as a template for *in vitro* transcription.

To generate the plasmid for recombinant protein expression, *ZmCPK32* was amplified using the primer pair 32GFP-F/32ET-R and cloned into pET-32a (Novagen) with *Bam*HI and *Hind*III restriction enzymes.

For transient expression in tobacco pollen, the promoter of the gene *Zm13* (accession number DQ312298) expressed specifically in maize pollen was amplified using the primers Zm13-F and Zm13-R. The plasmid pRTLZM13GFP was generated by replacing the 35S CaMV region of pRTL2NGFP with the *Zm13* promoter using *Hinc*II and *Xho*I [24]. Then, the coding regions of ZmCPK32 without a stop codon was amplified using the primers 32GFP-F and 32GFP-R and cloned into the vector pRTLZM13GFP using *Bam*HI and *Xba*I. To delete the N-terminal acylation site, an N-terminal truncated fragment, ZmCPK32ΔN, was amplified using the primers ΔN32GFP-F and 32GFP-R and introduced into pRTLZM13GFP with *Bam*HI and *Xba*I. To express the kinase-deficient (KD) mutant, ZmCPK32KD, the mutation (Asp^230^ to Asn^230^) was generated by overlapping PCR using the primer pairs 32GFP-F/32KD-R, 32KD-F/32GFP-R, and 32GFP-F/32GFP-R. Then, the PCR fragment of ZmCPK32KD was cloned into pRTLZM13GFP using *Bam*HI and *Xba*I. The constitutively active (CA) construct was generated by truncating ZmCPK32 at the junction between the kinase and autoinhibitory domains using the primers 32GFP-F and 32CA-R. Finally, the corresponding CA fragment was inserted into pRTLZM13GFP using *Bam*HI and *Xba*I. All generated plasmids were confirmed based on sequence analysis.

### qRT-PCR

To analyze *ZmCPK32* expression in various organs, root, stem, leaf, and sheath were sampled at the flare opening stage, while tassel, cob, silk, and anther were collected during flowering. The developing embryo and endosperm were harvested at 13, 15, 17, 19, 21, and 23 days after pollination (DAP). For analysis at different male gametophyte developmental stages, the developing tassels were collected according to their length: 0–5, 5–10, 10–15, 15–20, 20–25, and 25–30 cm. Total RNA was isolated with plant TRIzol reagent (Invitrogen) and treated with DNase I (Promega) to eliminate DNA contamination. First strand cDNA was synthesized using M-MLV reverse transcriptase (Promega). To examine the expression of Zm*CPK32*, the gene-specific primers 32-Q-RT-F and 32-Q-RT-L were designed in the 3′-untranslated region. *ZmActin1* expression was used as an internal control. The primer sequences for qRT-PCR are listed in S1 Table. The total PCR reaction volume was 20 μL, and consisted of 10 μL of SYBR qPCR premix (TaKaRa), 0.25 μM of each of the forward and reverse primers, and 1 μL of cDNA preparation. qRT-PCR was performed using a 7500 Real-Time PCR System (Applied Biosystems). PCR was run under the following conditions: initial denaturation at 95°C for 30 s, followed by 40 cycles of amplification at 95°C for 5 s and 60°C for 34 s. A melting curve analysis was performed to ensure specific amplification. The PCR products were also verified by electrophoresis and sequencing. Data were calculated with the ΔΔCT method using the ABI 7500 software. Three technical replicates were performed for each sample, and representative data from three independent experiments with similar results are presented.

### mRNA *in situ* hybridization

Stamens were sampled before anthesis and fixed in formaldehyde–acetic acid–ethanol, dehydrated in an ethanol series, cleared with xylene, and embedded in paraffin as described previously [24, 25]. Paraffin-embedded tissues were cut into 10-μm-thick sections and placed on slides coated with poly-L-lysine (Sigma). Digoxigenin (DIG)-labeled antisense and sense RNA probes were transcribed from either *Xho*I- or *Xba*I-digested pEASY-Zm*CPK32ISH* using either T7 (antisense) or SP6 (sense) RNA polymerase (Roche), respectively. *In situ* hybridization was performed as described previously [25]. After the enzyme-catalyzed color reaction, an insoluble blue precipitate was observed. Slides were visualized with a Zeiss Axioscop 40 microscope and photographed with an Mrc5 camera (Zeiss).

### Recombinant protein expression and kinase assay

The recombinant Trx-ZmCPK32 protein was expressed in *Escherichia coli* Rosetta 2 (DE3) pLysS (Novagen) via isopropyl β-D-1-thiogalactopyranoside induction and purified using Ni-NTA His·Bind Resin (Novagen). To examine kinase activity, 0.5 μg of Trx-ZmCPK32 and 2 μg of histone III were added into the reaction mixture containing kinase buffer (25 mM Tris-HCl pH 7.5, 10 mM MgSO_4_, and 1 mM dithiothreitol) in the presence of either 1 mM CaCl_2_ or 2 mM egtazic acid (EGTA). All reactions were initiated by adding 100 μM of ATP at 30°C for 30 min and terminated by adding sodium dodecyl sulfate-polyacrylamide gel electrophoresis (SDS-PAGE) loading buffer. The proteins were separated using 12.5% (w/v) SDS-PAGE and stained with Pro-Q Diamond phosphoprotein gel stain (Invitrogen) as previously described [26, 27] or Coomassie Brilliant Blue. Phosphorylated protein was visualized using a Molecular Imager FX (Bio-Rad) under 532 nm excitation and a 580 nm bandpass emission filter.

### Particle bombardment-mediated transient expression in tobacco pollen

Tobacco pollen grains were transformed using a PDS-1000/He particle delivery system (Bio-Rad). Pollen grains were collected from freshly dehisced anthers and stored at -80°C until use. For each bombardment, 4 mg of pollen was suspended in 200 μL of pollen germination medium (1 mM CaCl_2_, 1 mM Ca(NO_3_)_2_, 1 mM MgSO_4_, 0.01% H_3_BO_3_, and 10% sucrose, pH 6.5) [28]. Then, the pollen suspension was placed on a piece of pre-wetted nylon membrane for bombardment. Microprojectiles were prepared by coating 1 mg of gold particles (1.0 μm) with 3 μg of plasmid, and were used for two bombardments per sample. Bombardment was performed with a 28-inch-Hg vacuum using a 1100-psi rupture disk and a 6-cm target distance. Pollen grains were washed from the membrane with 1.6 mL of germination medium onto a 35-mm Petri dish immediately after bombardment and cultured on a rotary shaker at 26°C at 100 rpm. After germination for 4 h, transformed pollen grains showing green fluorescence were visualized and photographed under a Zeiss LSM 700 confocal microscope. Three biological replicates were conducted, and more than 50 transformed pollen grains were scored for the germination rate and pollen tube length per replicate. Statistical analysis was performed using one-way analysis of variance with Tukey’s test (P < 0.05).

## Results

### Phylogenetic relationship between ZmCPKs and their orthologs with known functions in pollen tube development

**Fig 1:**
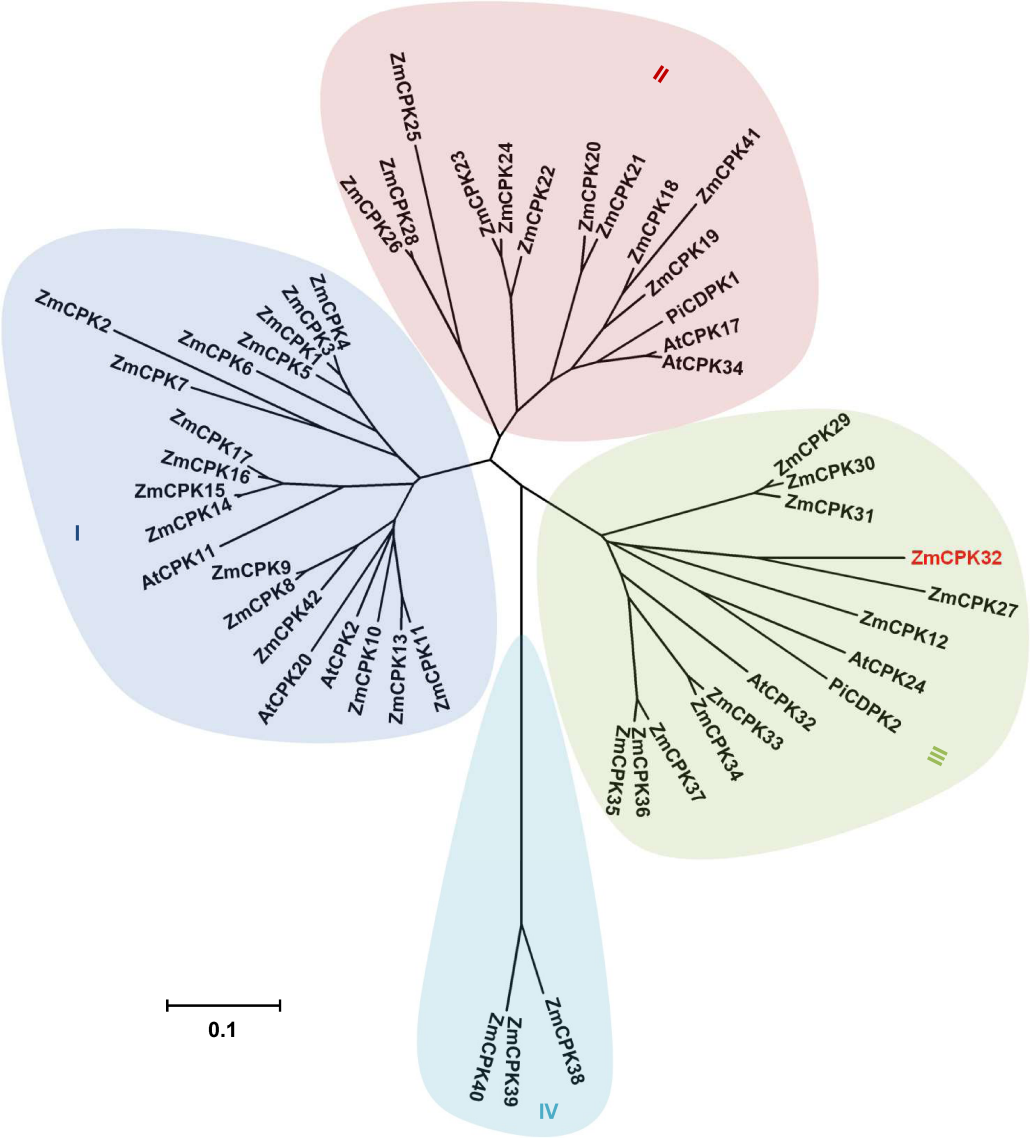
Phylogenetic analysis of maize CPKs. A phylogenetic tree between CPKs from maize and those functionally identified in pollen tube growth was established using the neighbor-joining method in MEGA ver. 4.0 software. The four subgroups are labeled as I, II, III, and IV, and ZmCPK32 is highlighted in red. The accession numbers used for the phylogenetic analysis are listed in the methods. The scale bar corresponds to a distance of 10 changes per 100 amino acid positions.

To identify maize CPKs potentially associated with pollen tube development, we analyzed the phylogenetic relationship of ZmCPKs with *Arabidopsis* and *Petunia inflata* CPKs involved in the regulation of pollen tube development. The maize CPK family was originally identified as containing 35 to 40 isoforms [29, 30], although a recent study resized this family to 39 members [31]. These analyses identified *CPK* genes in maize, but some isoforms were not included in overlapping studies. Therefore, we collected 42 nonredundant *CPK* isoforms from previous reports and designated them according to the nomenclature given by Kong *et al*. [30], with the added genes numbered consecutively (Table 1). Then, we analyzed the phylogenetic relationship between maize CPKs and those functionally associated with pollen tube growth in *Arabidopsis* and *P*. *inflata* (Fig 1). The ZmCPK members could be clustered into four subgroups, which was also observed for their orthologs in *Arabidopsis* and rice. The CPKs associated with pollen tube development were classified in subgroups I, II, and III.

**Table 1:** Characteristics of *CPK* members in maize.

Name	Identifier	Chromosome	Exons	Amino acid	Subgroup
*ZmCPK01*	GRMZM2G314396_T01	2	7	547	I
*ZmCPK02*	GRMZM2G040743_T01	1	8	540	I
*ZmCPK03*	GRMZM2G321239_T01	10	7	556	I
*ZmCPK04*	D87042^a^	1	-	554	I
*ZmCPK05*	GRMZM2G081310_T01	4	7	562	I
*ZmCPK06*	GRMZM2G347047_T01	4	2	488	I
*ZmCPK07*	GRMZM2G032852_T02	1	8	544	I
*ZmCPK08*	GRMZM2G027351_T01	5	8	584	I
*ZmCPK09*	GRMZM2G121228_T01	1	8	580	I
*ZmCPK10*	GRMZM2G353957_T01	3	5	646	I
*ZmCPK11*	GRMZM2G028926_T01	1	7	608	I
*ZmCPK12*	GRMZM2G097533_T01	3	7	438	III
*ZmCPK13*	GRMZM2G320506_T01	5	7	620	I
*ZmCPK14*	GRMZM2G035843_T01	4	8	508	I
*ZmCPK15*	GRMZM2G047486_T03	2	8	510	I
*ZmCPK16*	GRMZM2G347226_T01	10	8	515	I
*ZmCPK17*	GRMZM2G463464_T01	3	8	515	I
*ZmCPK18*	GRMZM2G167276_T01	3	7	510	II
*ZmCPK19*	GRMZM2G340224_T01	8	8	613	II
*ZmCPK20*	GRMZM2G365815_T01	2	7	552	II
*ZmCPK21*	GRMZM2G472311_T01	4	6	581	II
*ZmCPK22*	GRMZM2G058305_T01	8	8	539	II
*ZmCPK23*	GRMZM2G025387_T01	8	9	530	II
*ZmCPK24*	GRMZM5G856738_T03	3	7	524	II
*ZmCPK25*	GRMZM2G112057_T01	10	8	539	II
*ZmCPK26*	GRMZM2G154489_T01	7	9	531	II
*ZmCPK27*	GRMZM2G080871_T02	7	7	511	III
*ZmCPK28*	GRMZM2G168706_T01	2	9	531	II
*ZmCPK29*	GRMZM2G030673_T01	8	7	541	III
*ZmCPK30*	GRMZM2G088361_T01	6	7	540	III
*ZmCPK31*	GRMZM2G311220_T01	8	7	536	III
*ZmCPK32*	GRMZM2G332660_T01	4	5	568	III
*ZmCPK33*	AC210013.4_FGT014	5	8	538	III
*ZmCPK34*	GRMZM2G104125_T01	1	8	535	III
*ZmCPK35*	AC233871.1_FGT003	6	8	539	III
*ZmCPK36*	GRMZM2G028086_T01	7	8	539	III
*ZmCPK37*	GRMZM2G099425_T01	2	8	539	III
*ZmCPK38*	GRMZM2G365035_T01	2	12	512	IV
*ZmCPK39*	GRMZM2G157068_T01	5	12	522	IV
*ZmCPK40*	GRMZM2G053868_T01	4	12	522	IV
*ZmCPK41*	AC203294.3_FGT001	8	6	464	II
*ZmCPK42*	GRMZM2G012326_T01	2	9	685	I

^a^The GenBank accession number.

### Expression profiles of the *ZmCPK* gene family

**Fig 2:**
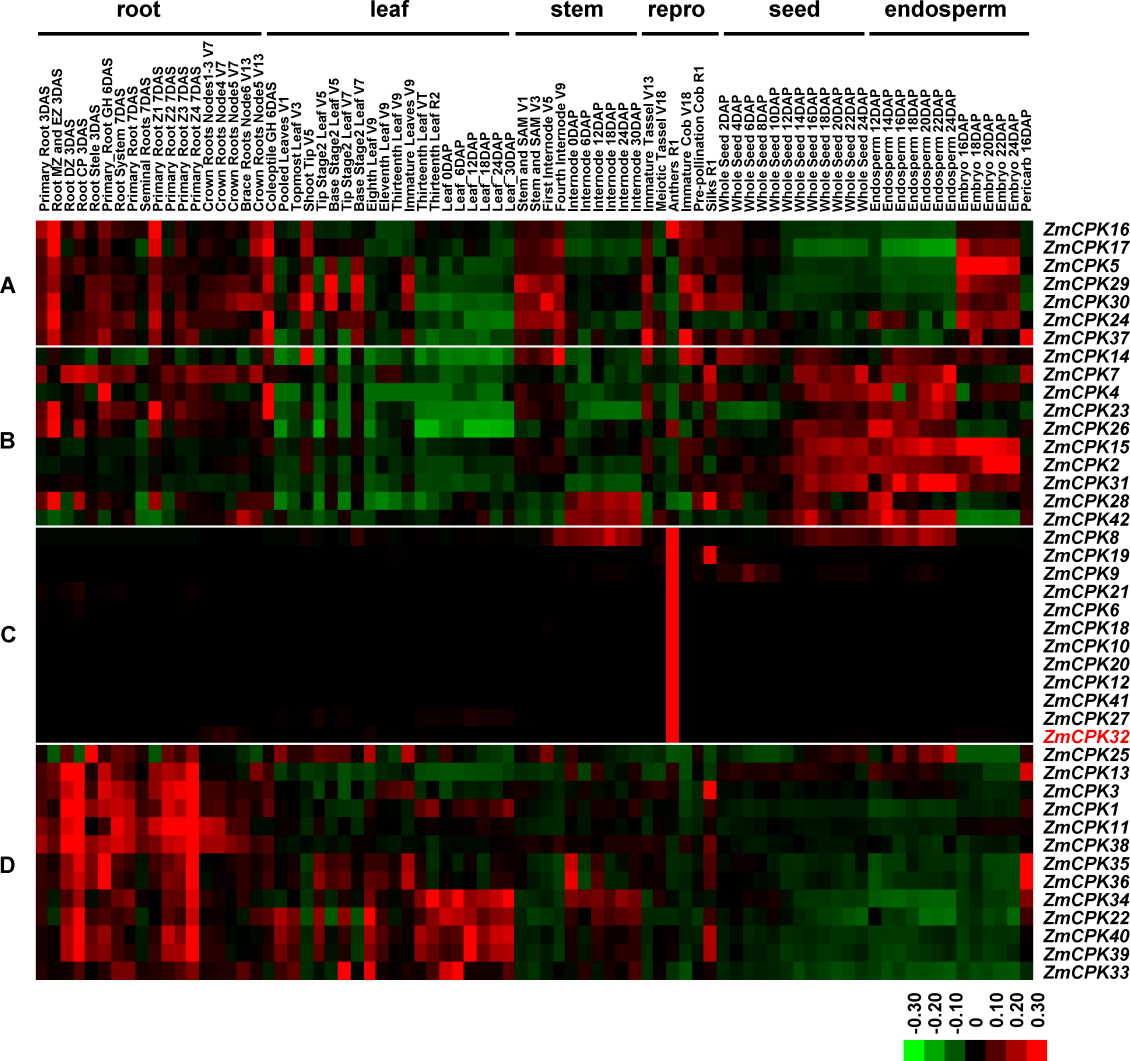
Expression patterns of maize *CPK* genes in different tissues. A heatmap was generated to portray the expression levels of maize *CPK* genes in 79 distinct tissues, including root, leaf, stem, reproductive organs (repro), seed, and endosperm at various developmental stages. *ZmCPK32* is highlighted in red and *ZmCPKs* were clustered into four groups (A–D) according to their expression patterns. The scale representing the expression intensity is shown at the bottom.

To determine the putative functions of ZmCPKs, we analyzed the expression patterns of maize *CPK* genes using published RNA sequencing (RNA-seq) data for 79 different tissues covering all developmental stages of maize [23]. According to the generated heatmap (Fig 2), the expression profiles of maize *CPK* genes could be classified into four groups (A–D). Group A genes accumulated in roots, vegetative leaves, and stems, as well as in cobs and embryos, suggesting they may have roles associated with cell proliferation and elongation. Group B genes were minimally detected in leaves, but highly expressed in endosperms at 12–24 DAP, indicating that they may function in starch synthesis. Group C genes were predominantly expressed in anther, suggesting that they may participate with either male gametocyte formation or pollen tube germination and elongation. Group D genes were preferentially expressed in roots, suggesting that they may be essential for root development.

### Isolation of *ZmCPK32* from flowering tassels

ZmCPK32 was found in group C (i.e., predominantly expressed in anthers) and was closely related to PiCDPK2 (DQ147912) and AtCPK24 (At2g31500), which have been reported to regulate pollen tube development [14, 18]. Both the gene expression pattern and phylogenetic relationship suggest that *ZmCPK32* may be associated with pollen tube growth regulation; therefore, we cloned this gene for further investigation. ZmCPK32 contains a highly variable N-terminal region, a conserved kinase domain, an autoinhibitory domain, and a CaM-LD with four EF-hands (S1 Fig). Moreover, there are potential lipid modification sites at the N-terminus, suggesting that ZmCPK32 may localize on membranes. The protein sequence alignment revealed that ZmCPK32 shares a high amino acid sequence similarity with other CPKs that mediate pollen tube growth, while the N-terminal domain is distinct, indicating that they may have different substrate specificities.

### *ZmCPK32* expression in pollen during pollination

**Fig 3:**
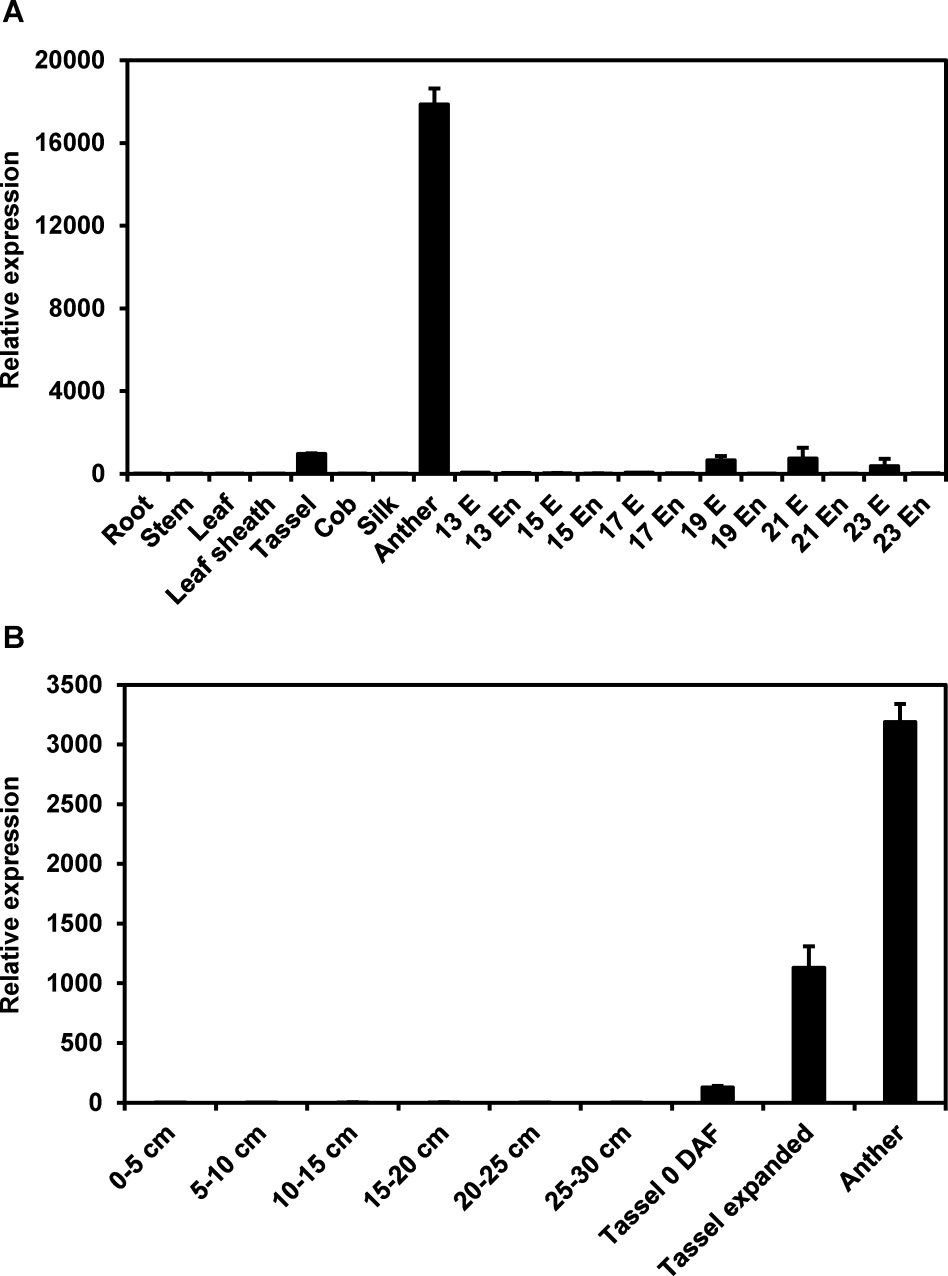
*ZmCPK32* accumulates predominantly in anthers during shedding. (A) Expression of *ZmCPK32* in different organs and developing seeds. The transcript levels of *ZmCPK32* in vegetative tissues, including root, stem, leaf, and leaf sheath, reproductive tissues such as tassel, cob, silk, and anther, and the embryo (E) and endosperm (En) at the indicated days after pollination (13–23 DAP) were examined by qRT-PCR. (B) Expression profiles of *ZmCPK32* in the developing tassel. The whole tassel development process was divided into eight stages, including the immature tassel stage, according to its length, and tassels were collected on the day of flowering (0 DAF) and branch expansion. *ZmActin1* expression was used as an internal control, and the error bars indicate standard deviation.

Since the RNA-seq results revealed that *ZmCPK32* was highly expressed in anthers, qRT-PCR was used to further examine *ZmCPK32* transcripts throughout reproductive development (Fig 3A). Abundant accumulation of *ZmCPK32* was detected in anthers, while weak expression could be detected in tassels and developing embryos (19–23 DAP). To determine *ZmCPK32* expression during gametophyte development, growing tassels were divided into six stages according to length. *ZmCPK32* expression was dramatically upregulated when tassels started shedding and the transcripts continued to accumulate until tassels were fully expanded (Fig 3B).

**Fig 4:**
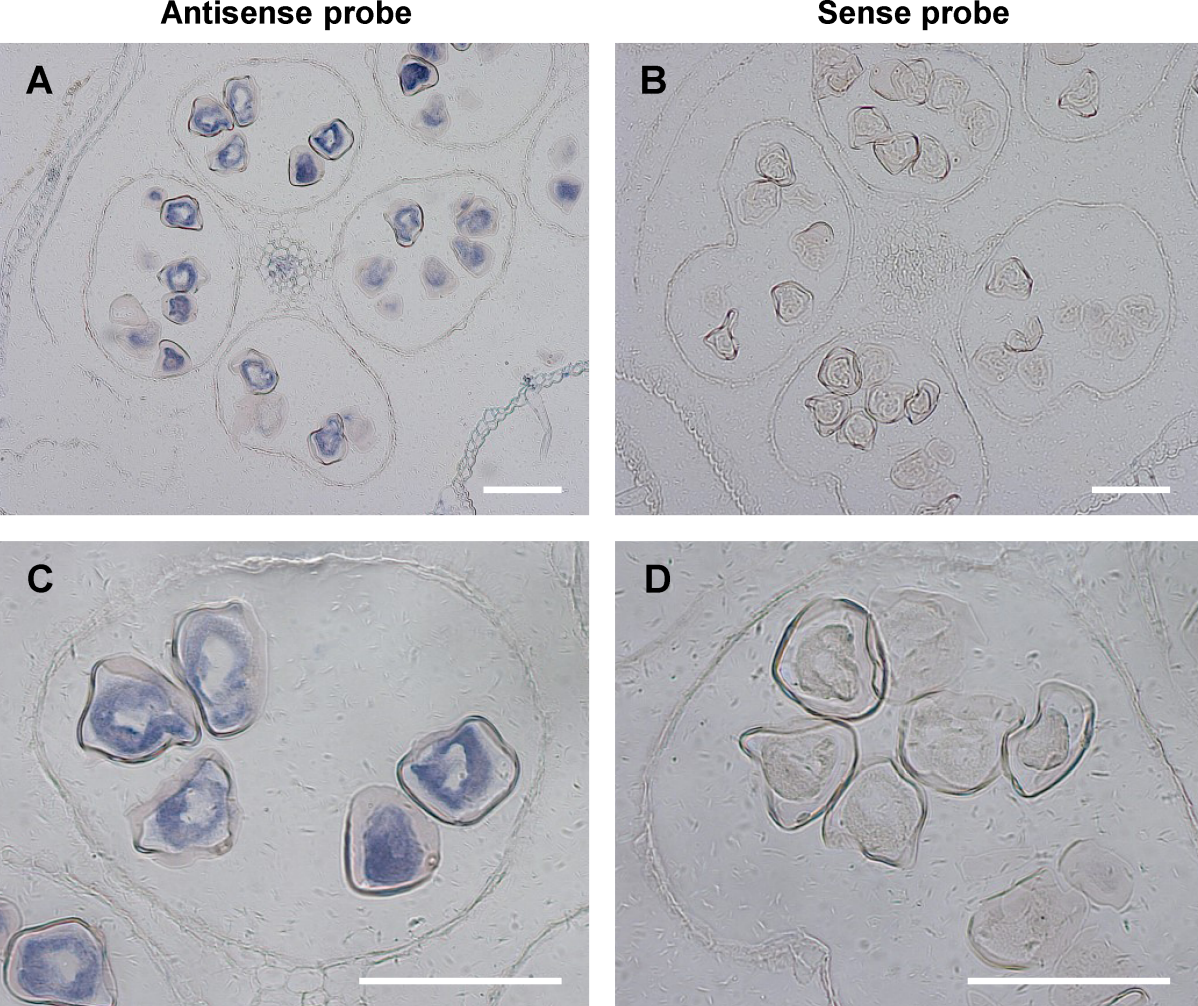
*In situ* hybridization showing pollen-specific expression of *ZmCPK32*. (A and C) *ZmCPK32* transcripts were detected in pollen in transverse sections of anthers hybridized with antisense probes. (B and D) No signal was observed in sections hybridized with sense probes. Panels C and D are enlargements of A and B, respectively. The scale bar lengths correspond to 100 μm.

Because *ZmCPK32* was highly expressed in anthers during shedding, *in situ* hybridization was employed to study the histological localization of *ZmCPK32*. Transverse sections of mature anthers were made (Fig 4). The transcripts of *ZmCPK32* were mainly detected in pollen using gene-specific DIG-labeled antisense RNA probes (Fig 4A and 4C), and no signal was observed in sections probed with labeled sense RNA probes (Fig 4B and 4D). These results suggest that *ZmCPK32* may have a role in regulating pollen tube growth, which correlates with its expression specificity.

### The kinase activity of ZmCPK32

**Fig 5:**
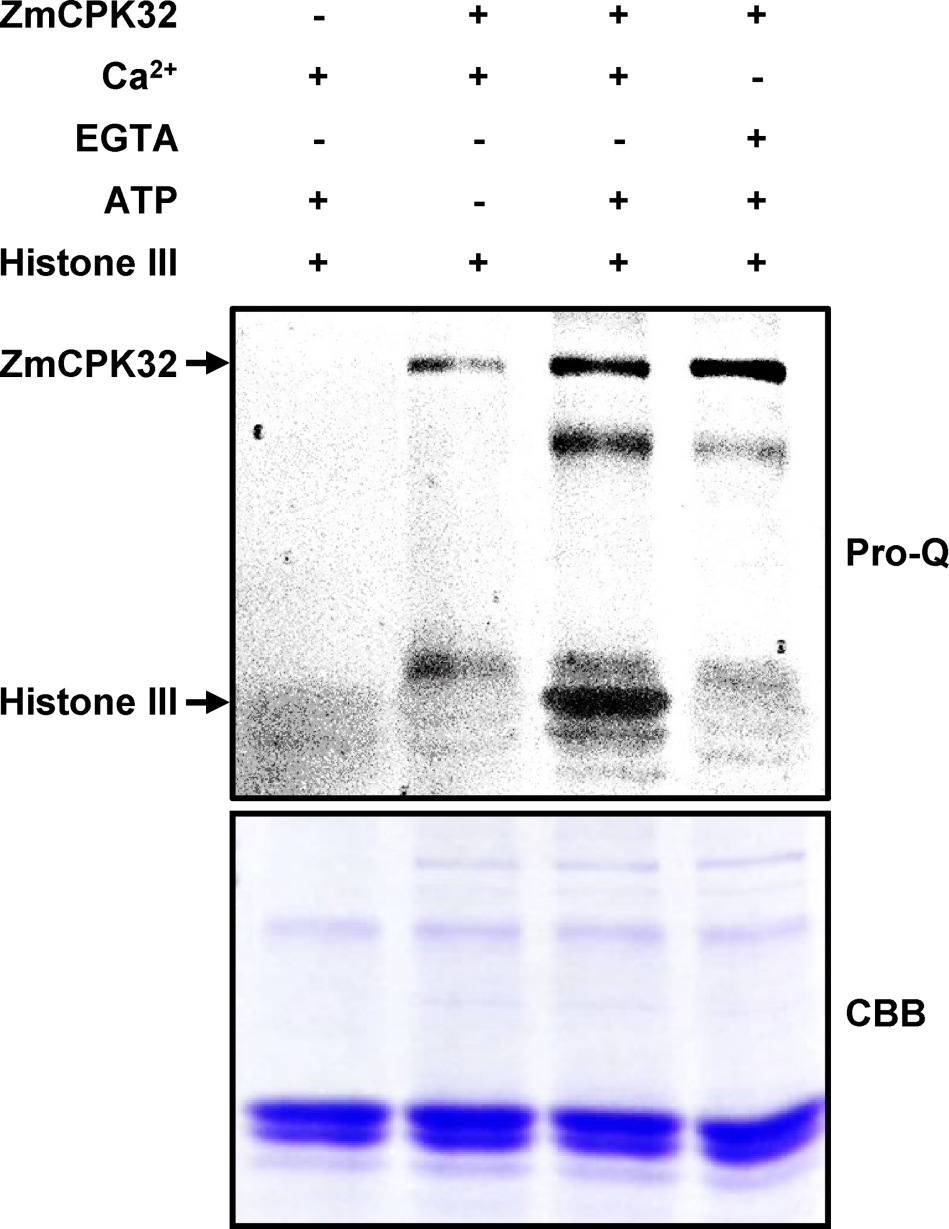
*In vitro* kinase assay of ZmCPK32. Purified Trx-ZmCPK32 recombinant protein was subjected to kinase reactions in the presence of 1 mM Ca^2+^ or 2 mM EGTA, and histone III was used as a substrate. Following SDS-PAGE, the gel was stained with Pro-Q Diamond phosphoprotein gel stain to visualize phosphorylated proteins (Pro-Q) or Coomassie Brilliant Blue (CBB). Auto-phosphorylated Trx-ZmCPK32 and phosphorylated histone III are indicated by arrows.

To determine the kinase activity of ZmCPK32, the full-length open reading frame was expressed in *E*. *coli* as a thioredoxin (Trx) fusion protein and the soluble recombinant protein was purified with the 6×His tag. The substrate phosphorylation activities of ZmCPK32 were analyzed using a phosphoprotein staining approach [26, 27]. ZmCPK32 phosphorylated histone III in the presence of 1 mM Ca^2+^, which was inhibited by 2 mM EGTA, suggesting that ZmCPK32 has CPK activity (Fig 5).

### Inhibition of pollen tube growth by transient expression of *ZmCPK32*

**Fig 6:**
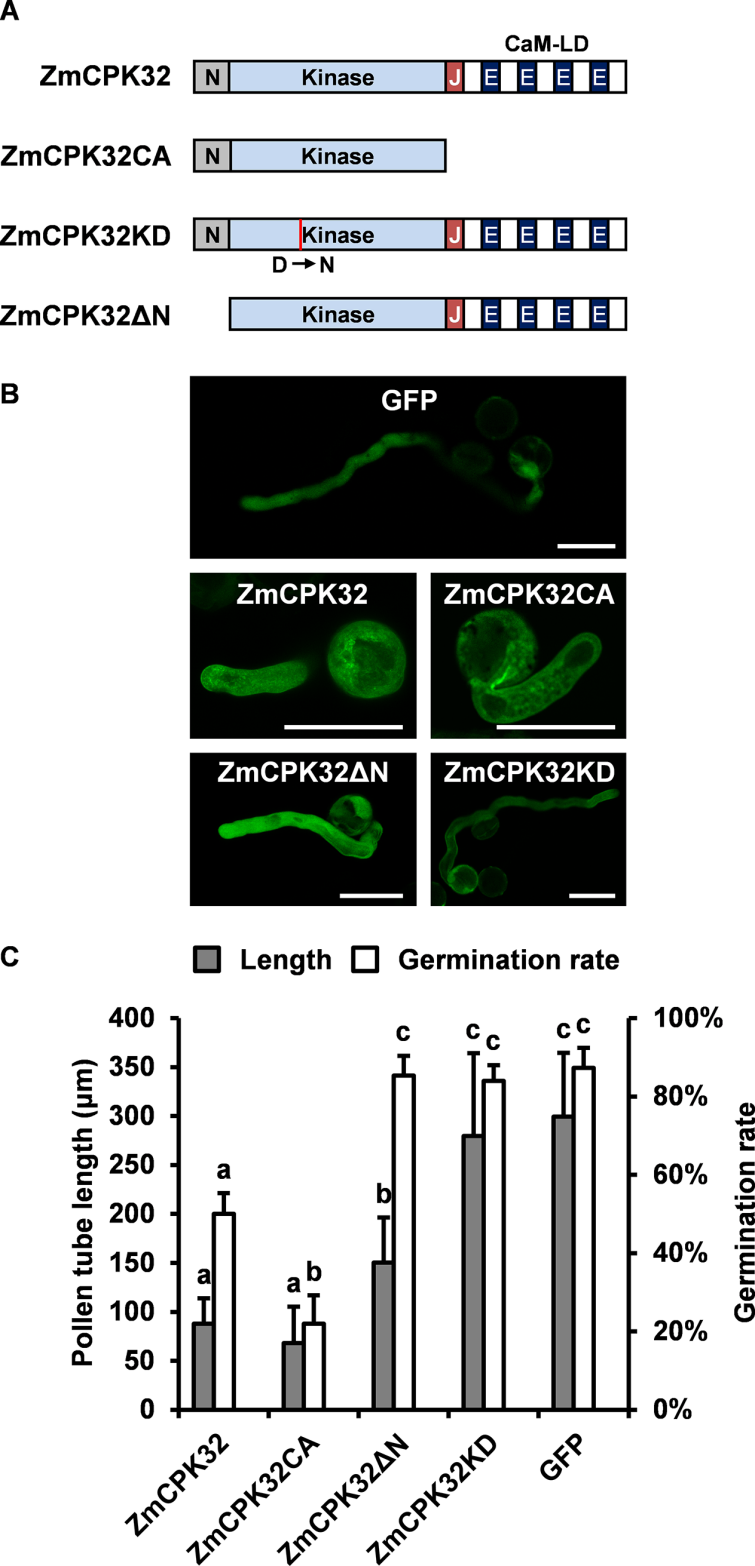
Transient expression of ZmCPK32 suppresses tobacco pollen tube growth. (A) Schematic diagram showing the structures of fusion proteins used for pollen transient expression. Wild-type, constitutively active (CA), kinase domain-deficient (KD), and N-terminal-deleted (ΔN) variants of ZmCPK32 were generated as GFP fusion proteins and introduced into tobacco pollen grains via microparticle bombardment. The red line in ZmCPK32KD indicates the mutation (Asp^230^ to Asn^230^) in the kinase domain. (B) Representative pollen tubes transformed with the indicated plasmid. Images were taken 4 h after germination. The scale bars represent 50 μm. (C) Quantitative analysis of pollen tube growth phenotypes. The germination rate and pollen tube length were measured from more than 50 transformed pollen grains after culturing *in vitro* for 4 h. Different lowercase letters represent significant differences (P < 0.05) according to Tukey’s test. The error bars indicate standard deviations.

Since *ZmCPK32* transcripts were predominantly detected in pollen during shedding, we speculate that *ZmCPK32* may be involved in the regulation of pollen tube germination and/or elongation. To investigate the function of *ZmCPK32* in pollen tube growth, the ZmCPK32-GFP fusion protein-expressing construct was generated (Fig 6A) and introduced into tobacco pollen grains using microprojectile bombardment. For pollen expression, the maize *Zm13* promoter was applied [32, 33]. Compared with the GFP-expression control, ZmCPK32-GFP-expressing pollen showed a reduced germination rate and pollen tube length (Fig 6B and 6C). To determine whether pollen tube growth inhibition depended on the kinase activity of ZmCPK32, CA and KD variants of ZmCPK32 were generated and introduced into tobacco pollen grains. ZmCPK32CA-GFP accumulation further suppressed the germination rate, although pollen tube elongation showed no significant difference compared with ZmCPK32-GFP-transformed pollen. However, pollen expressing ZmCPK32KD-GFP exhibited normal germination and pollen tube elongation (Fig 6B and 6C), suggesting that the inhibition of pollen tube growth depends on the kinase activity of ZmCPK32.

### ZmCPK32 localization at the plasma membrane and internal membrane compartments in pollen tube and effects on pollen tube elongation

**Fig 7:**
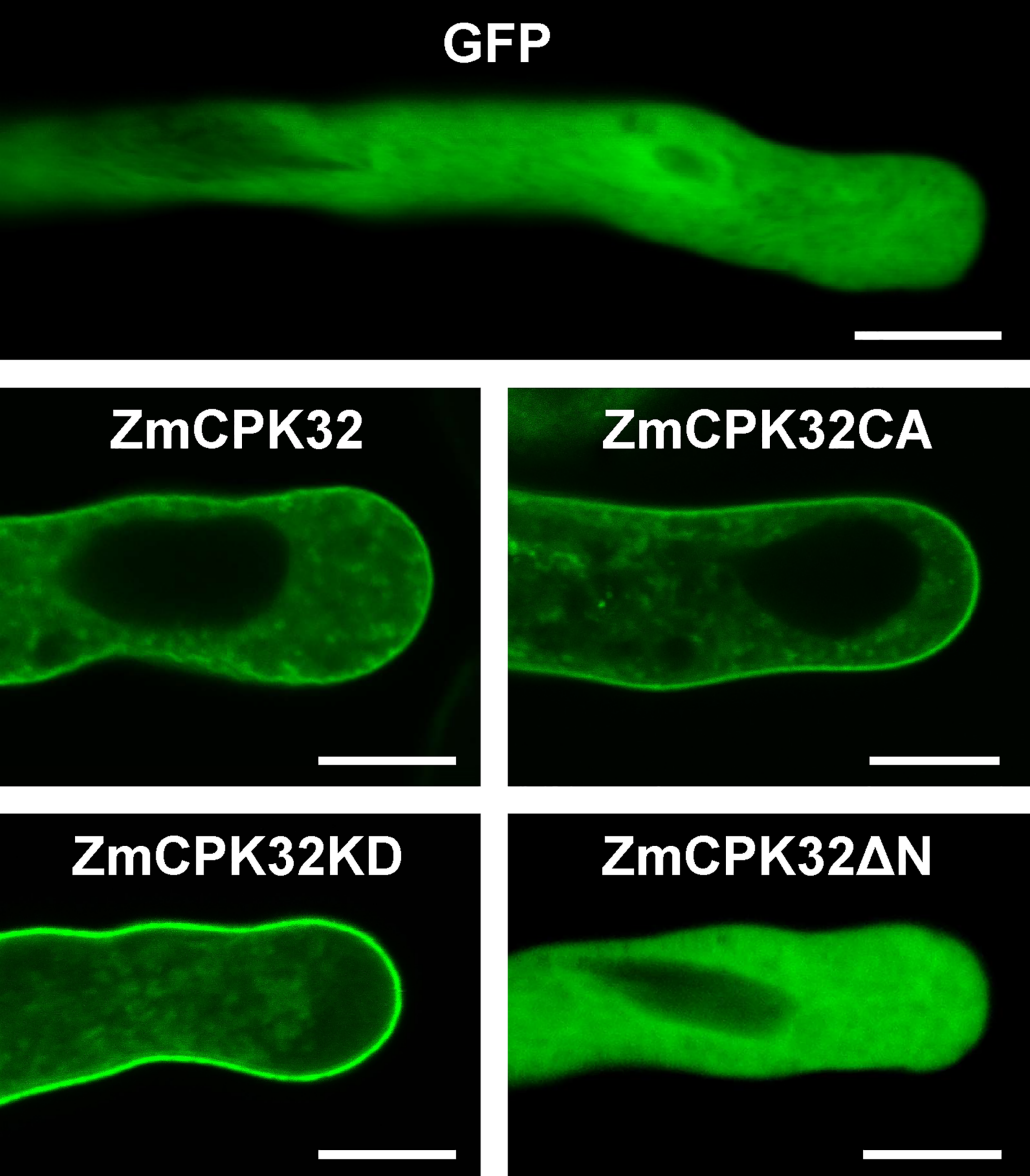
ZmCPK32 targets both the plasma membrane and punctate internal membrane compartments in the pollen tube via the N-terminal domain. The full length, constitutive active (CA), kinase deficient (KD), and N-terminal-deleted versions of ZmCPK32 were fused at the C-terminus with GFP and expressed in tobacco pollen under the control of the maize *Zm13* promoter. Images were taken 4 h after germination. The scale bars represent 10 μm.

N-terminal acylation is essential for CPK membrane anchoring. ZmCPK32 possessed both a potential myristoylation site (i.e., Gly residue at position 2) and palmitoylation site (i.e., Cys residue at position 4) based on a similarity search using NMT (The MYR Predictor, http://mendel.imp.ac.at/myristate/SUPLpredictor.htm) and CSS-Palm ver. 3.0 software (http://csspalm.biocuckoo.org/online.php), respectively. Thus, the subcellular localization of ZmCPK32 was observed in pollen tubes. The GFP fusion proteins of ZmCPK32, ZmCPK32CA, and ZmCPK32KD were localized to both the plasma membrane and punctate structures throughout the pollen tube (Fig 7). To examine whether N-terminal lipid modification determined the membrane localization, the acylation-deleted mutant ZmCPK32ΔN-GFP was transiently expressed in pollen grains. Fluorescence signals were detected mainly in the cytosol, similar to the GFP control (Fig 7), suggesting that the N-terminal sequence is necessary for the membrane localization of ZmCPK32 in the pollen tube.

Finally, ZmCPK32ΔN-GFP was transiently expressed in pollen to test whether the inhibitory effects on pollen tube growth relied on membrane localization. ZmCPK32ΔN-GFP expression enabled normal pollen germination, but caused a weak reduction in pollen tube elongation (Fig 6B and 6C). This suggests that membrane localization of ZmCPK32 is essential for its function in pollen tube extension.

## Discussion

In plants, CPK-regulated Ca^2+^ signaling has been implicated in many aspects of plant development, including as a response to environmental stresses and plant hormones, and in reproductive development [34]. Many *CPK* families have been identified and functionally characterized in plants based on well-assembled genome sequences. There are reportedly 34 CPK isoforms in *Arabidopsis* [35, 36], 31 members in rice [37–39], and 35–40 CPKs in maize [29–31], suggesting that CPKs are widespread in higher plants and encoded by a large multigene family.

Although the maize *CPK* gene family has been analyzed *in silico* in several studies, these genes were named under distinct criteria, and some isoforms have not been covered by overlapping phylogenetic and expression analyses [29–31]. Here, we systematically analyzed the *ZmCPK* family by collecting nonredundant maize CPKs and assigned the gene names according to the criteria used by Kong *et al*. [30] with continuous numbering of added isoforms [31]. As an advantage, this naming convention provides an effective method to trace the phylogenetic relationships among CPK members, since they are phylogenetically classified with nearly consecutive names: ZmCPK1–11, 13–17, and 42 in subgroup I; ZmCPK18–26, 28, and 41 in subgroup II; ZmCPK12, 27, and 29–37 in subgroup III; and ZmCPK38–40 in subgroup IV. In addition, we analyzed the expression profiles of *ZmCPK* genes using the latest published maize gene expression atlas with 79 tissues covering all developmental stages [23]. Eleven maize *CPK* isoforms (*ZmCPK6*, *9*, *10*, *12*, *18*–*21*, *27*, *32*, and *41*) were predominantly expressed in the anther, suggesting that they may participate in male gametophyte development.

To date, only four members of maize CPKs have been functionally clarified. For example, the stimulated gene expression and increased kinase activity of ZmCPK11 (ZmCPK15 in this work) was observed in damaged leaves, suggesting that ZmCPK11 is involved in the wounding response [40]. Further research found that the wounding activation of ZmCPK11 was jasmonate- and linolenic acid-dependent [41]. In addition, ZmCPK11 was reported to be involved in abscisic acid (ABA)-induced antioxidant defense, since ABA and H_2_O_2_ treatments activated ZmCPK11, which further stimulated ZmMPK5 to regulate antioxidant enzymes [42]. Moreover, overexpression of *ZmCPK4* (D87042) in *Arabidopsis* enhanced the ABA sensitivity in seed germination and seedling growth and the expression of several ABA-responsive genes, suggesting that ZmCPK4 may be a positive regulator of ABA signaling [43]. ZmCPK1 (ZmCPK7 in this work) was reported to be a negative regulator of cold stress responses, where transient expression of *ZmCPK1* in leaf protoplasts repressed the expression of a cold-responsive gene, *Zmerf3*, and *ZmCPK1*-overexpressing transgenic *Arabidopsis* plants exhibited reduced tolerance to freezing temperatures [31]. These studies revealed the functions of maize CPKs under stress conditions and in phytohormone signal transduction, such as mechanical wounding, cold tolerance, and ABA signaling. However, among the 11 genes of group C predominantly expressed in anthers, only one CPK, ZmCPK20 (GRMZM2G365815), has been functionally characterized in promoting pollen tube germination and growth [9]. One study found that 26 proteins altered the phosphorylation status in maize pollen upon germination for 1 h, indicating that phosphorylation is required for pollen tube growth regulation [44]. Moreover, a comparative proteome and phosphor-proteome analysis found that many CPKs were enriched as phosphorylated proteins in maize pollen, suggesting that CPKs may serve as an essential switch in regulating maize pollen tube growth [22].

In this study, *ZmCPK32* specifically accumulated in pollen during shedding, suggesting that it may be associated with pollen tube development. In attempting to elucidate the physiological function of ZmCPK32, wild-type, KD, and CA variants were expressed in tobacco pollen grains as GFP fusion proteins. Overexpression of ZmCPK32-GFP and ZmCPK32CA-GFP suppressed both the germination and extension of the pollen tube, while transient expression of ZmCPK32KD-GFP had no effect on pollen tube growth. These results suggest that kinase activity is essential for the function of ZmCPK32 in regulating pollen tube growth. Moreover, ZmCPK32CA showed a more severe negative effect on pollen germination than wild-type kinase, suggesting that the cortical growth point-specific activation of ZmCPK32 by Ca^2+^ is essential for the regulation of appropriate germination. We found that ZmCPK32 localized at both the plasma membrane and internal membrane compartments in the pollen tube (Fig 7), which contributed partly to its function, since cytosol-localized ZmCPK32ΔN-GFP expression had no effect on germination, but weakly reduced the pollen tube length.

Compared with several functionally characterized CPKs that are essential for pollen tube growth, overexpression of ZmCPK32 generated negative effects. Considering the phylogenetic relationship, expression data, and known functions, we speculate that CPKs within different subgroups may have distinct roles in pollen tube development. The *ZmCPK* genes predominantly expressed in anthers were mainly assigned to three subgroups: ZmCPK6, 9, and 10 in subgroup I; ZmCPK18–21, and 41 in subgroup II; and ZmCPK12, 27, and 32 in subgroup III. Interestingly, CPKs with reported functions in pollen tube growth regulation were also assigned to these subgroups: AtCPK2 and 20 in subgroup I; AtCPK17, 34, and PiCDPK1 in subgroup II; and AtCPK24, 32, and PiCDPK2 in subgroup III. Moreover, the above CPKs in subgroups II and III formed compact clusters, suggesting that they may have different roles between groups but share closely related functions within each cluster.

In contrast to AtCPK17 and 34, which are positive regulators of pollen tube growth [15], AtCPK24 was phosphorylated by AtCPK11 and negatively regulated inward K^+^ movement and pollen tube elongation [18]. Similarly, inhibited pollen tube extension was observed when PiCDPK2 was overexpressed [14] and disrupted polar growth associated with increased Ca^2+^ concentrations at the pollen tube tip was observed when AtCPK32 was overexpressed in tobacco pollen [45]. These findings suggest that increasing the accumulation of *CPK*s predominantly expressed in pollen results in negative effects on pollen tube growth. As a possible explanation, CPKs have dedicated roles or are involved in feedback pathways that regulate pollen tube growth, and their expression must be strictly controlled.

Functional similarity also correlated with the subcellular localization of these CPKs to an extent. AtCPK17 and 34 targeted the plasma membrane of the pollen tube, while both plasma membrane and weak punctate structure signals were observed for AtCPK32, indicating potential internal membrane localization [17]. Differences in subcellular localization were also observed for two CPKs in *Petunia*. PiCDPK1 targeted the plasma membrane, while PiCDPK2 was localized in punctate internal compartments throughout the pollen tube [14], further identified as peroxisomes [46]. In our study, a similar punctate localization pattern was observed, and *ZmCPK32* overexpression inhibited pollen tube elongation, suggestive of similar localizations and functions between PiCDPK2 and ZmCPK32. In accordance with their different subcellular localizations, subgroup II (i.e., AtCPK17/34 and PiCDPK1) and subgroup III (i.e., PiCDPK2 and ZmCPK32) CPKs exhibited distinct functions in regulating pollen tube growth, indicating a potential link between subcellular localization and function.

In summary, we characterized mature pollen specifically expressing *ZmCPK32*, which acts as a negative regulator in pollen tube growth. Ca^2+^-regulated kinase activity was essential for ZmCPK32 function. ZmCPK32 was localized to the plasma membrane and unidentified punctate internal membrane compartments via N-terminal acylation, and appropriate localization of ZmCPK32 was necessary for its function in pollen tube extension. Further investigations are needed to reveal the nature of internal membrane localization and explore the substrates of this kinase.

## Supporting information

S1 Fig Amino acid sequence alignment of ZmCPK32 with CPKs that regulate pollen tube growth. The accession numbers and protein sequences used for the multiple alignment are listed in the methods. Highly conserved domains are underlined. The dots indicate gaps introduced to maximize the alignment. The light- and dark-shaded backgrounds indicate partial and entire similarity among residues, respectively.
(TIF)

S1 Table Primers used in this study. (XLSX)
