# Supplementary figures and images for: A calcium-dependent protein kinase, ZmCPK32, specifically expressed in maize pollen to regulate pollen tube growth
Source: PLoS One. 2018 May 29;13(5):e0195787. doi: 10.1371/journal.pone.0195787 (PMC5973587; doi:10.1371/journal.pone.0195787)

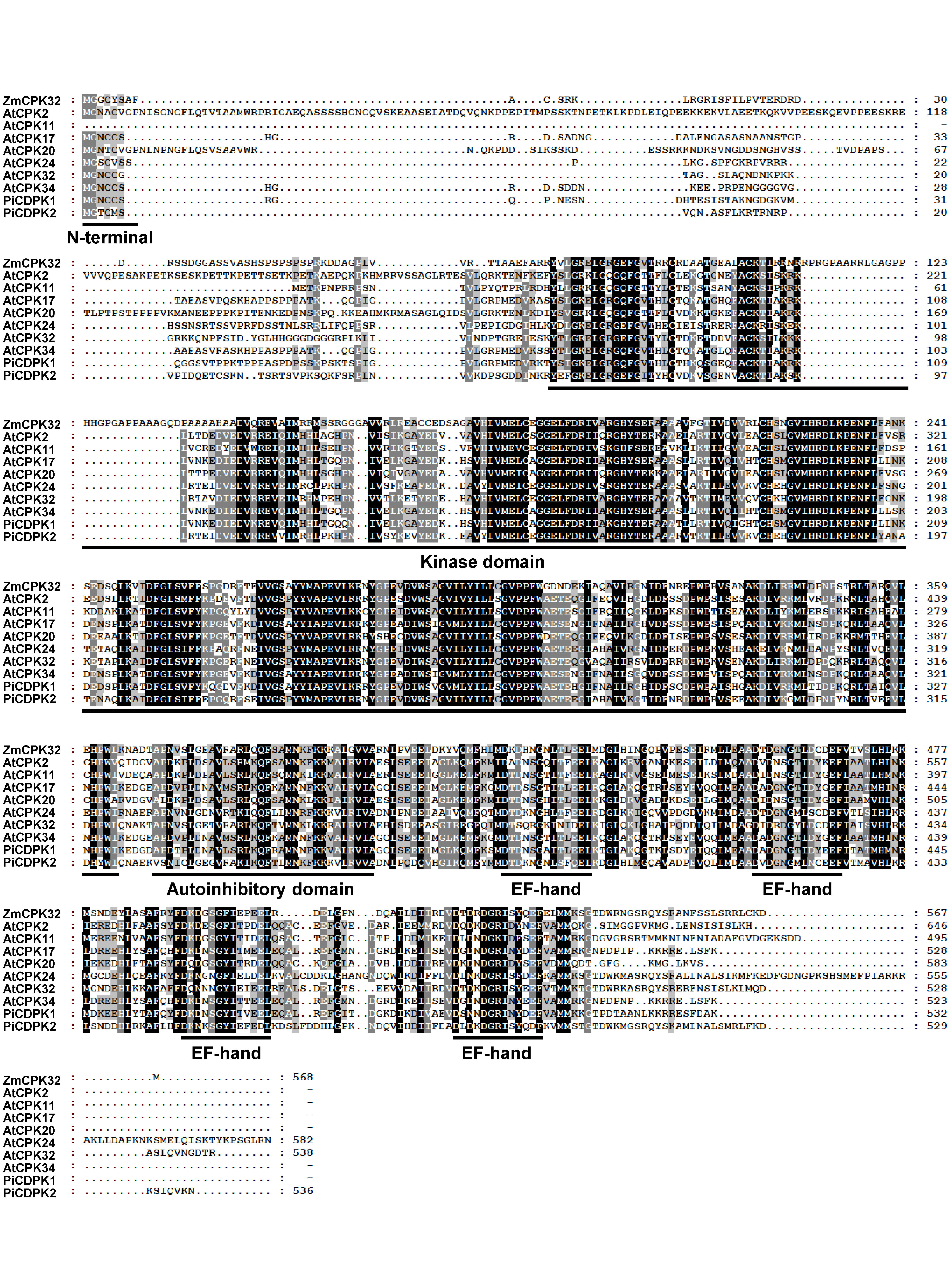

Supplement: S1 Fig — The accession numbers and protein sequences used for the multiple alignment are listed in the methods. Highly conserved domains are underlined. The dots indicate gaps introduced to maximize the alignment. The light- and dark-shaded backgrounds indicate partial and entire similarity among residues, respectively. (TIF) [file pone.0195787.s001.tif]
